# New Discorhabdin B Dimers with Anticancer Activity from the Antarctic Deep-Sea Sponge *Latrunculia biformis*

**DOI:** 10.3390/md18020107

**Published:** 2020-02-11

**Authors:** Fengjie Li, Dorte Janussen, Deniz Tasdemir

**Affiliations:** 1 GEOMAR Centre for Marine Biotechnology (GEOMAR-Biotech), Research Unit Marine Natural Products Chemistry, GEOMAR Helmholtz Centre for Ocean Research Kiel, Am Kiel-Kanal 44, 24106 Kiel, Germany; fli@geomar.de; 2 Senckenberg Research Institute and Natural History Museum, Senckenberganlage 25, D-60325 Frankfurt, Germany; dorte.janussen@senckenberg.de; 3 Faculty of Mathematics and Natural Sciences, Kiel University, Christian-Albrechts-Platz 4, 24118 Kiel, Germany

**Keywords:** *Latrunculia biformis*, discorhabdin B dimer, deep-sea sponge, Antarctica, anticancer activity, ECD spectroscopy

## Abstract

*Latrunculia* sponges represent a rich source of discorhabdin-type pyrroloiminoquinone alkaloids, a few of which comprise a dimeric structure. The anticancer-activity-guided isolation of the *n*-hexane subextract of the Antarctic deep-sea sponge *Latrunculia biformis* yielded the known compound (−)-(1*R*,2*R*,6*R*,8*S*,6’*S*)-discorhabdin B dimer (**1**) and two new derivatives, (−)-(1*S*,2*R*,6*R*,8*S*,6’*S*)-discorhabdin B dimer (**2**) and (−)-(1*R*,2*R*,6*R*,8*S*,6’*S*)-16’,17’-dehydrodiscorhabdin B dimer (**3**). The chemical structures of compounds **1**–**3** were elucidated by means of HR-ESIMS, NMR, [α]_D_, ECD spectroscopy, and a comparison with the previously reported discorhabdin analogs. Compounds **1** and **2** showed significant in vitro anticancer activity against the human colon cancer cell line (HCT-116), with IC_50_ values of 0.16 and 2.01 µM, respectively. Compared to monomeric discorhabdins, dimeric discorhabdins are very rare in Nature. This study adds two new discorhabdin dimers (**2** and **3**) to this small pyrroloiminoquinone subfamily. This is also the first report of compound **1** as a natural product and the first assessment of its in vitro anticancer activity.

## 1. Introduction

*Latrunculia* is the largest genus of the marine sponge family Latrunculiidae Topsent (Class Demospongiae Sollas, Order Poecilosclerida Topsent) [1,2]. The members of this genus are predominately distributed in cold-water regions of the Southern Hemisphere [3,4]. Continuing reports of pyrroloiminoquinone-type alkaloids with strong anticancer activity from *Latrunculia* sponges have been the main driving force for in-depth chemical analyses of this genus [5,6,7,8,9,10,11,12]. So far, *Latrunculia* sponges represent one of the major reservoirs of pyrroloiminoquinone-type alkaloids. More than 40 pyrroloiminoquinone alkaloids belonging to the subclasses tsitsikammamines, makaluvamines, and discorhabdins have been reported from this genus, the discorhabdins being the largest and the most diverse [5,8,13,14]. Discorhabdins are a unique class of nitrogenous pigments possessing a characteristic tetracyclic pyrido[2,3-*h*]pyrrolo[4,3,2-*de*]quinoline core bound to a cyclohexanone substituent at the spiro center C-6 [5]. Discorhabdins lend themselves for various types of substitutions, such as bromination at C-2 or C-4, which is common in discorhabdins. Some discorhabdins bear a heptacyclic backbone through further intramolecular cyclization via a bond formation between C-2 and N-18 and/or possess a thioether bridge between C-5 and C-8 [6,7,15]. The broad-spectrum anticancer activity of discorhabdins against various cancer cell lines, e.g., HCT-116 (human colon cancer cell line), P388 (murine leukemia cell line), and KB (human oral cancer cell line) have been reported [5,16]. Compared to monomeric discorhabdins, dimeric discorhabdins are highly uncommon. So far, only five natural discorhabdin dimers ((+)-discorhabdin W, (−)-discorhabdin W, (+)-16a,17a-dehydrodiscorhabdin W, (−)-16a,17a-dehydrodiscorhabdin W, and (−)-28,29-dihydrodiscorhabdin B dimer) that are linked to each other with a sulfide or disulfide bond have been reported and all derive from *Latrunculia* sp. collected from New Zealand or Australia [11,17,18]. In addition, the discorhabdin B dimer has been reported as an artifact that formed after the storage of discorhabdin B [19]. Notably, the discorhabdin dimers exhibit promising anticancer activities in the same magnitude as the monomeric discorhabdins [11,17].

In a recent study, we reported new discorhabdin monomers from the MeOH subextract of the Antarctic deep-sea sponge *L. biformis* [14]. In the continuation of the chemical investigation of this sponge, we have now focused on its *n*-hexane-soluble portion. Guided by in vitro anticancer activity against the human colon cancer cell line (HCT-116), we herein report three dimeric discorhabdins, the known compound (−)-(1*R*,2*R*,6*R*,8*S*,6’*S*)-discorhabdin B dimer (**1**) and two of its new derivatives, (−)-(1*S*,2*R*,6*R*,8*S*,6’*S*)-discorhabdin B dimer (**2**) and (−)-(1*R*,2*R*,6*R*,8*S*,6’*S*)-16’,17’-dehydrodiscorhabdin B dimer (**3**) from the *n*-hexane subextract of *L. biformis*. This study outlines the bioactivity-guided isolation and structure elucidation of compounds **1**–**3**, and in vitro anticancer activity determination of **1** and **2**.

## 2. Results

### 2.1. Purification and Structure Elucidation

**Figure 1:**
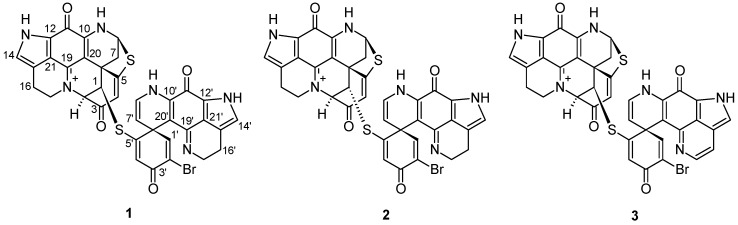
Chemical structures of compounds **1**–**3**.

As described previously [14], the sponge material was desalted with water, and extracted by methanol and dichloromethane, respectively. The combined organic extract was partitioned between MeOH and *n*-hexane. The C18 SPE fractionation of the *n*-hexane subextract yielded nine subfractions (H1-H9). Repeated RP-HPLC of the active subfractions H4 and H5 afforded three minor compounds, **1**–**3** (Figure 1).

The structure of compound **1** was identified by comparison of its HRMS and 1D NMR data (Table 1 and Table 2) with those reported for discorhabdin B dimer [19]. Furthermore, we revised the chemical shifts of several quaternary carbon atoms, C-15, C-21, C-15’, and C-21’, on the basis of full 2D NMR data analysis (Table 2). The HMBC correlations between H-16/C-21 and H-17/C-15 (Supplementary Figure S3), led to the assignment of the ^13^C signals at *δ*_C_ 119.4 and *δ*_C_ 122.8 to C-15 and C-21, respectively, which were ascribed vice versa in the literature [19]. The chemical shifts of C-15’ and C-21’ were also revised in the same fashion. The [α]${}_{D}^{20}$ value of compound **1** (−144, *c* 0.01 MeOH) had the same sign and similar magnitude as the known compound (−)-(1*R*,2*R*,6*R*,8*S*,27*S*)-discorhabdin B dimer (−120, *c* 0.05 MeOH) [19]. Hence, compound **1** was characterized as the (−)-(1*R*,2*R*,6*R*,8*S*,6’*S*)-discorhabdin B dimer.

**Figure 2:**
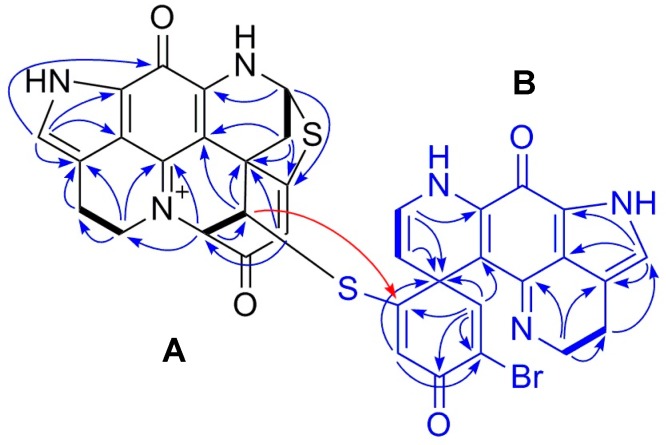
Key COSY (bold lines) and HMBC (arrows) correlations observed for compound **2**. The letters **A** and **B** indicate each discorhabdin monomer.

Compound **2** was purified as a greenish TFA salt. The HR-ESIMS spectrum of **2** showed the quasi-molecular ions at *m/z* 747.0487/749.0476 [M]^+^ (Supplementary Figure S12) in a ratio of approximately 1:1, indicating the presence of one bromine atom and hence generating the same molecular formula C_36_H_24_BrN_6_O_4_S_2_ as **1**. A comparison of the ^1^H and ^13^C NMR spectra of **2** with those of **1** (Table 1 and Table 2) revealed high similarity between these two compounds, indicating that **2** is a dimeric discorhabdin alkaloid. A detailed analysis of the 1D and 2D NMR data of **2** suggested the presence of five sp^3^ methylenes, three sp^3^ methenes, seven sp^2^ methenes, and 21 quaternary carbon signals belonging to two sp^3^ and 19 sp^2^ carbon atoms (Table 1 and Table 2). To facilitate the structure elucidation and interpretation of the NMR data of **2**, we assigned the letters **A** and **B** for each discorhabdin monomer (Figure 2). Characteristic ^1^H NMR resonances attributed to component **A** for a pyrroloiminoquinone moiety in the spectrum of **2** included those appeared at *δ*_H_ 7.09 (H-14), *δ*_H_ 5.58 (H-8), *δ*_H_ 3.91 (H_2_-17), *δ*_H_ 3.03 (H_2_-16), *δ*_H_ 2.95, and 2.76 (H_2_-7) (Table 1) [9,10,11,14]. The tetracyclic pyrroloiminoquinone core structure of component **A** was readily ascertained by the 2D COSY and HMBC correlations shown in Figure 2. In detail, the COSY cross-peak between H_2_-16 and H_2_-17 together with the observed HMBC correlations between H_2_-16/C-15, H_2_-17/C-15, H_2_-17/C-19, H-14/C-11, H-14/C-12, and H-14/C-21 established the tricyclic pyrrolo[4,3,2-*de*] quinoline moiety (Figure 2). The tetrahydropyridine ring could then be connected to the tricyclic system based on the COSY correlation between H_2_-7 and H-8 and the HMBC correlations between H_2_-7/C-20 and H-8/C-10, which establishes the tetracyclic pyrroloiminoquinone core structure of **A** (Figure 2). The 2D COSY cross-peak between H-1 and H-2, and the HMBC correlations between H-1/C-5, H-1/C-6, H-1/C-20, H-2/C-1, H-2/C-3, H-2/C-6, H-4/C-2, and H-4/C-6 supported the presence of a cyclohexanone substitution at the spiro center, C-6. The down-field shifted resonances of C-5 (*δ*_C_ 174.4), C-8 *(δ*_C_ 62.8), and H-8 (*δ*_H_ 5.58) indicated the presence of a sulfide bridge between C-5 and C-8 [9,10,11,14]. This assumption was further supported by a diagnostic HMBC correlation between H-8 and C-5 (Figure 2). HMBC correlations between H-2/C-17 and H-2/C-19 revealed a further intramolecular ring closure via a bond formation between C-2 and N-18 (Figure 2). The multiplicity and the down-field chemical shift of H-1 (*δ*_H_ 5.03, d, *J* = 2.6 Hz) and H-2 (*δ*_H_ 4.43, d, *J* = 2.6 Hz) indicated an electronegative substitution at C-1. Thus, **A** was elucidated as a C1-substituted discorhabdin L-type alkaloid.

The pyrroloiminoquinone moiety in component **B** was established in a similar fashion. The ^1^H NMR resonances at *δ*_H_ 7.23 (H-14’), *δ*_H_ 3.91 (H_2_-17’), and *δ*_H_ 2.97 (H_2_-16’) signified the existence of a pyrroloiminoquinone moiety in unit **B** (Table 1), which was further ascertained by the observed COSY and HMBC correlations shown in Figure 2. The COSY correlation between H-7’ (*δ*_H_ 4.82, d, *J* = 7.5 Hz) and H-8’ (*δ*_H_ 6.58, d, *J* = 7.5 Hz) and the observed HMBC correlations between H-7’/C-6’, H-7’/C-8’, H-7’/C-20’ and H-8’/C-6’, H-8’/C-7’, H-8’/C-10’ supported the ∆^7’(8’)^ unsaturation (Figure 2). Further HMBC correlations shown in Figure 2, namely between H-1’/C-2’, H-1’/C-3’, H-1’/C-5’, H-1’/C-6’, H-1’/C-20’ and H-4’/C-2’, H-4’/C-5’, H-4’/C-6’ indicated a cyclohexanone moiety at C-6’. Based on the similarity of the chemical shift of C-2’ (*δ*_C_ 124.4) to that of discorhabdin W, which contains a bromine atom at C-2’ (*δ*_C_ 125.1) [9], as well as the observation of an HMBC correlation between H-4’ and C-2’, the bromine atom was assigned to C-2’. The down-field shift of C-5’ (*δ*_C_ 164.7) and the HMBC correlation between H-1’/C-5’ completed the full structure elucidation of **B** and proved the presence of a thioether bridge between C-1 and C-5’ (Figure 2). Hence, the planar dimeric structure of **2** was confirmed.

**Figure 3:**
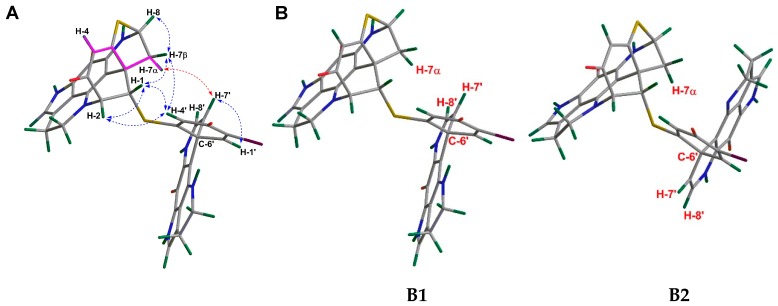
(**A**) Key NOE correlations drawn on a Chem3D optimized model of **2**. (**B**) Two proposed molecular models (**B1** and **B2**) of **2**.

The identification of the relative configuration of **2** was mainly based on its NOE data (Figure 3). Due to a further intramolecular ring closure formed by the sulfide bridge between C-5 and C-8, and the direct bond between C-2 and N-18, the unit A was configurationally restrained. This allows only one stable relative configuration at C-2, C-6, and C-8, implying that C-2/C-3, C-6/C-7, and C-8/S are *syn*-periplanar to each other (Figure 3A). The diastereotopic methylene resonances at *δ*_H_ 2.76 (dd, *J* = 1.5, 11.7 Hz) and 2.95 (dd, *J* = 3.6, 11.7 Hz) were assigned to have α and β orientations, respectively, according to the long-range COSY correlation (Supplementary Figure S10) between H-4 (*δ*_H_ 6.27 ppm) and H-7α (*δ*_H_ 2.76 ppm). This correlation indicated a planar ‘W’ arrangement [20], which is very common for many heptacyclic discorhabdins, e.g., discorhabdin D, discorhabdin L, and 1-acetyldiscorhabdin L [7,14,15]. The NOE correlation between H-1 and H-7β indicated the β-orientation of H-1, which was further supported by the NOE cross-peaks between H-1/H-4’, H-1/H-2, H-4’/H-7β, and H-4’/H-2 (Figure 3A). The relative configuration of the stereocenters in **B** (C-6’) was established by the NOE correlation between H-7’ and H-7α (Figure 3A). Since the relative configuration of unit **A** was established, two molecular models (**B1** and **B2**) that represented the two possible relative configurations at C-6’ were proposed (Figure 3B). Only **B1** allows a NOESY correlation between H-7’/H-7α, confirming the same relative configuration of C-6’ as in **1**. Thus, compound **2** was elucidated as an epimer of **1**, with an opposite configuration at C-1.

**Figure 4:**
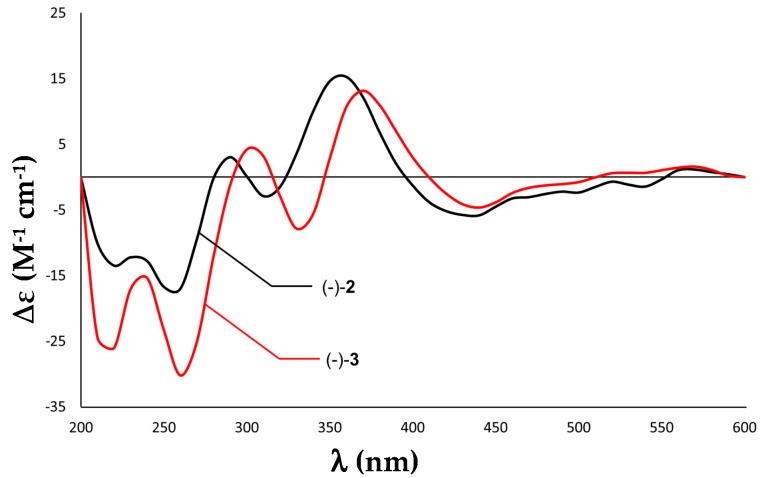
Experimental ECD spectra (MeOH) of the TFA salts of (−)-**2** (black) and (−)-**3** (red).

The absolute configuration of **2** was established by ECD spectroscopy. The ECD spectra of discorhabdin-type alkaloids with a stereogenic spiro center at C-6 are characterized by a high-amplitude Cotton effect (CE) in the 325–400 nm region, whereby positive and negative CEs in this region of the ECD spectra imply a β- and an α-orientation of C-5/C-6 bond, respectively, when the pyrroloiminoquinone backbone is drawn as unit **A** in Figure 1 [9,10,11,14]. Hence, the high-amplitude positive CE around 350 nm in the ECD spectrum of **2** signifies a β-oriented C-5/C-6 bond in unit **A** (Figure 4). Considering the assigned relative configuration and the same sign of its optical rotation ([α]${}_{D}^{20}$ −473, *c* 0.05, MeOH) as that of **1** ([α]${}_{D}^{20}$ −144, *c* 0.01 MeOH), **2** was established as a new compound, namely (−)-(1*S*,2*R*,6*R*,8*S*,6’*S*)-discorhabdin B dimer.

**Figure 5:**
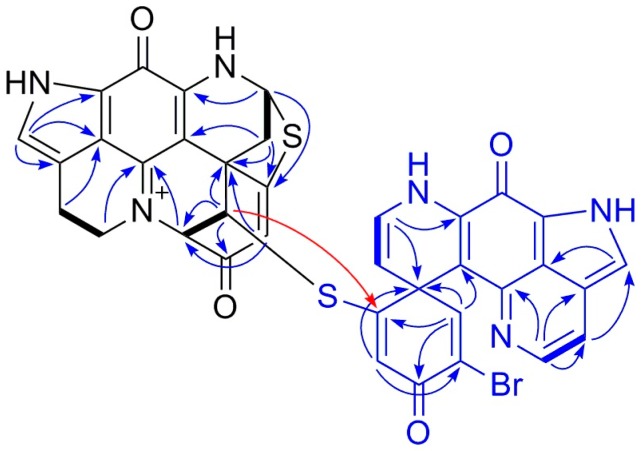
Key COSY (in bold) and HMBC (arrows) correlations observed for compound **3**.

Compound **3** was purified as an orange TFA salt. Its molecular formula was deduced as C_36_H_22_BrN_6_O_4_S_2_ from the molecular ions (*m/z* 745.0328/747.0319 [M]^+^) observed in its HR-ESIMS spectrum (Supplementary Figure S19). This indicated the presence of 29 degrees of unsaturation. A comparison of the 1D NMR data of **3** with those of **1** revealed a high resemblance between the two molecules. The major difference was the replacement of the two methylene resonances, namely H_2_-16’and H_2_-17’ with two olefinic protons in **3** (H-16’, *δ*_H_ 2.97 in **1** and *δ*_H_ 7.64 in **3**; H-17’ *δ*_H_ 3.91 in **1** and *δ*_H_ 8.19 in **3**), indicating that **3** is a new dehydro derivative of **1** (Table 1 and Table 2). The 2D structure of **3** was further confirmed by an in-depth inspection of its 2D NMR data shown in Figure 5. A ^1^H-^1^H COSY correlation between H-16’ and H-17’, coupled with the HMBC correlations between H-16’/C-14’, H-17’/C-16’, and H-17’/C-19’, confirmed the position of the double bond at ∆^16’(17’)^ (Figure 5). The carbonyl signals belonging to C-11 and C-11’ did not appear in the NMR spectra, which is a common case in many pyrroloiminoquinone alkaloids [11,12,14]. Our attempts to locate the two carbonyl carbons by using a high-resolution cryo NMR probe were unsuccessful, however, the structural homology was obvious based on the HRMS, 1D, and 2D NMR data and the comparison of the data with related compounds in the literature [18,19].

**Figure 6:**
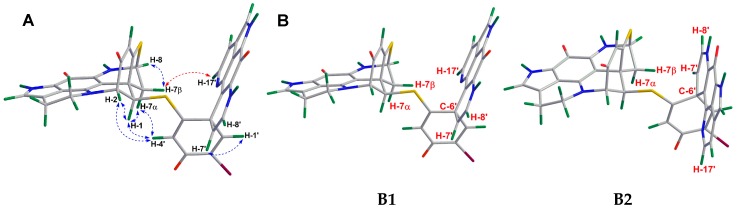
(**A**) Key NOE correlations drawn on a Chem3D optimized model of **3**. (**B**) Two proposed molecular models (**B1** and **B2**) of **3**.

The relative configuration of **3** was established in the same fashion as **2**. Based on its NOESY spectrum, C-2, C-6, and C-8 in **A** had the same relative configurations as in **1** and **2** (Figure 6A). NOE correlations between H-1/H-7α, H-1/H-4’, H-1/H-2, H-2/H-4’, and finally between H-4’/H-7α confirmed the α-orientation of H-1, which is the same in **1** (Figure 6A). For **B**, the relative configuration at C-6’ was established by the NOE correlation between H-17’ and H-7β (Figure 6A). Two molecular models (**B1** and **B2**) representing two possible relative configurations at C-6’ were proposed, with only **B1** allowing a spatial correlation (H-17’/H-7β). Hence, the relative configuration of **3** was established, as shown in Figure 6A. The high-amplitude positive Cotton effect around 375 nm in the ECD spectrum of **3** indicates a β-oriented C-5/C-6 bond in **A** (Figure 4). Hence, the absolute configuration of **3** was identified as 1*R*,2*R*,6*R*,8*S*,6’*S*, which was further supported by its ECD spectrum (Figure 4). The specific rotation value of **3** ([α]${}_{D}^{20}$ −239, *c* 0.1 MeOH) confirmed its structure as a (−)-(1*R*,2*R*,6*R*,8*S*,6’*S)*-16’,17’-dehydrodiscorhabdin B dimer.

### 2.2. Bioactivity Evaluation

Due to a supply issue, only compounds **1** and **2** were tested against the human colon cancer cell line (HCT-116) and the human keratinocyte cell line (HaCaT) (Table 3). Limited by its minute amount, compound **3** was not tested in any cell line. Both **1** and **2** showed promising anticancer activity against HCT-116 cells with IC_50_ values of 0.16 and 2.01 μM, respectively (Table 3). However, they also inhibited the growth of HaCaT cells with similar IC_50_ values (0.56 and 4.69 μM for **1** and **2**, respectively, Table 3), indicating their general toxicity.

## 3. Discussion

In a previous study, we isolated six monomeric discorhabdins from the MeOH subextract of the Antarctic deep-sea sponge *L. biformis* [14]. In the present study, we were able to isolate three more lipophilic dimeric discorhabdin alkaloids from the *n*-hexane subextract of the same sponge and determine their chemical structures despite their very minor amounts. Compared to discorhabdin monomers, dimeric discorhabdins are relatively rare. Out of over 40 discorhabdin alkaloids that have been reported so far, only five represent a dimeric structure [9,10,11,17,18]. Notably, discorhabdin B dimer (**1**) has been reported as an artifact of discorhabdin B after a long-time storage and its bioactivity has never been studied [19]. This is the first isolation of **1** as a natural product on its own. This is also the first assessment of **1**’s in vitro anticancer activity. We exclude the possibility that the discorhabdin B dimer (**1**) can be an artifact of the isolation procedure, as we were able to detect the compound in the crude sponge extract.

The structural elucidation of discorhabdins is hampered by their highly strained backbone, commonly found heteroatoms (N, O, S) and halogenation (e.g., Br), and a high degree of unsaturation. This explains the limited numbers of hydrogen atoms in the discorhabdin structure. In the case of dimeric discorhabdins, the overlapping resonances from two similar discorhabdin units make the structural elucidation even more difficult. In the present study, structures of compounds **1**–**3** were unambiguously elucidated by an in-depth analysis of their spectroscopic data (HR-ESIMS, NMR, [α]_D_) and a comparison with the previously reported discorhabdins. Compound **2** was elucidated as a C-1 epimer of the known compound **1**. To our knowledge, this the first report of C-1 epimeric discorhabdins. Compound **3** was characterized as the dehydro derivative of **1**, with the only difference contributed by the ∆^16’(17’)^ unsaturation in unit **B**. With the exception of this difference, the 2D structure of unit **A** remained the same for both compounds **1** and **3**. The configuration of the stereogenic centers within **2** and **3** were secured by their NOESY and ECD spectroscopy data. The ECD spectroscopy was first employed in the structural elucidation of discorhabdins by Grkovic et al. in 2008 [9]. Since then, this technique has become particularly attractive and useful for confirming the absolute configuration of discorhabdins because of their conformationally rigid structures, strong UV absorption and circular dichroism properties [10,11,13,21]. So far, ECD spectroscopy is also one of the most effective techniques to secure the absolute configurations of discorhabdins-type alkaloids with a large number of stereogenic centers.

Discorhabdin-type alkaloids are characterized by their wide spectrum of in vitro anticancer activities [5,16]. Notably, the anticancer potency of the dimeric discorhabdins is similar to the monomeric discorhabdin with IC_50_ values at a submicromolar level. For example, both enantiomeric pairs of discorhabdins W and 16a,17a-dehydrodiscorhabdin W exhibit very strong inhibition towards the P388 murine leukemia cell line with IC_50_ values ranging from 0.084 to 0.45 µM [9,10,11,17]. More recently, the 28,29-dihydro discorhabdin B dimer with promising activity against the HCT-116 cell line was reported as a novel inhibitor of HIF-1α/p300 protein–protein interactions [18]. In the current study, both compound **1** and its new epimer **2** were found to exhibit anticancer potency toward HCT-116 with IC_50_ values of 0.16 and 2.01 µM, respectively. The fact that compound **1** displays approximately 13 times lower IC_50_ value than **2** against the HCT-116 cells may indicate that *R* stereochemistry at C-1 is favored for anticancer activity. In comparison to its monomeric unit (−)-discorhabdin L (IC_50_ = 0.94 µM against HCT-116) [14], the potency of compound **1** is almost six times higher. However, when tested against the non-cancerous human keratinocyte cell line HaCaT, both compounds **1** and **2** show toxicity (Table 3), suggesting their low selectivity. Further isolation from natural resources coupled with the chemical synthesis of monomeric and dimeric discorhabdin derivatives may shed light on their structure–anticancer activity relationship and the reduction of their toxicity.

## 4. Materials and Methods

### 4.1. General Procedures

The specific rotation of compounds **1**–**3** were measured on a Jasco P-2000 polarimeter (Jasco, Pfungstadt, Germany). The FT-IR spectra were recorded using a PerkinElmer Spectrum Two FT-IR spectrometer (PerkinElmer, Boston, MA, USA). The ECD spectra were run in MeOH on a J-810 CD spectrometer (Jasco, Pfungstadt, Germany). The NMR spectra were obtained on a Bruker AV 600 spectrometer (600 and 150 MHz for ^1^H and ^13^C NMR, respectively, Bruker^®^, Billerica, MA, USA) equipped with 5.0 mm Shigemi tube (SHIGEMI, Co., LTD., Tokyo, Japan). The residual solvent signals were used as internal references: *δ*_H_ 3.31/*δ*_C_ 49.0 ppm (MeOD), and *δ*_H_ 2.50/*δ*_C_ 39.51 ppm (DMSO-*d_6_*). 4-Dimethyl-4-silapentane-1-sulfonic acid (DSS) served as the internal standard. HR-ESIMS was recorded on a micrOTOF II-High-performance TOF-MS system (Bruker^®^, Billerica, MA, USA) equipped with an electrospray ionization source. Solid-phase extraction (SPE) was performed on the Chromabond SPE C18 column cartridges (6 mL/2000 mg, Macherey-Nagel, Duren, Germany). HPLC separations were performed on a VWR Hitachi Chromaster system (VWR International, Allison Park, PA, USA) consisting of a 5430 diode array detector (VWR International, Allison Park, PA, USA), a 5310 column oven, a 5260 autosampler, and a 5110 pump combined in parallel with a VWR Evaporative Light Scattering Detector (ELSD 90, VWR International, Allison Park, PA, USA). The eluents used for HPLC separations were H_2_O (A) and MeCN (B). Routine HPLC separations were performed on an analytical synergi column (250 × 4.6 mm, Phenomenex, Torrance, CA, USA) and an analytical C6-Phenyl column (250 × 4.6 mm, Phenomenex, Torrance, CA, USA). The organic solvents used for HPLC isolation were HPLC grade (ITW Reagents, Darmstadt, Germany). The water used was MilliQ-water produced by Arium^®^ Water Purification Systems (Sartorius, Germany).

### 4.2. Sponge Material, Extraction, and Isolation

The details of the collection and extraction of the sponge material have been reported previously [14]. Lyophilization and extraction of the sponge material followed by a liquid-liquid partition of the crude extract between MeOH and *n*-hexane yielded the polar MeOH and nonpolar *n*-hexane subextracts [14]. The *n*-hexane subextract (H, 120 mg) was eluted with a step gradient MeOH/H_2_O mixture (0% to 100%) on a Chromabond C18 SPE cartridge to afford 9 subfractions (H1–H9). The active subfractions H4 (14.9 mg) and H5 (22.0 mg) were further fractionated individually on a Chromabond C18 SPE cartridge eluted with a MeOH/H_2_O (0.1% TFA, from 60% to 100%) mixture to furnish further subfractions (H4.1 to H4.5; H5.1 to H5.5). The RP-HPLC chromatography of H4.1 (2.1 mg) equipped with an analytical synergi column (gradient of H_2_O/MeCN from 75:25 to 70:30 in 10 min; 70:30, 10–15 min, with 0.1% TFA, flow 1.0 mL/min) yielded compound **2** (0.2 mg, *t*_R_ 10.8 min). The RP-HPLC chromatography of H4.3 (3.0 mg) on the same column (gradient of H_2_O/MeCN from 74:26 to 69:31 in 10 min; 69:31, 10–17 min, with 0.1% TFA, flow 1.0 mL/min) afforded compound **1** (0.3 mg, *t*_R_ 12.2 min). The RP-HPLC chromatography of H5.2 (6.5 mg) on an analytical C6-Phenyl column (gradient of H_2_O/MeOH from 56:44 to 41:59 in 24 min, with 0.1% TFA, flow 1.0 mL/min) gave compound **3** in a pure state (0.1 mg, *t*_R_ 14.8 min).

*(−)-(1R,2R,6R,8S,6’S)-Discorhabdin B dimer* (**1**): green film; [α]${}_{D}^{20}$ = −144 (*c* 0.01, MeOH); ^1^H NMR (CD_3_OD, 600 MHz) and ^13^C NMR (CD_3_OD, 150 MHz, extracted from HSQC and HMBC spectra) Table 1 and Table 2; HR-ESIMS *m/z* [M]^+^ 747.0487 (calcd. for C_36_H_24_BrN_6_O_4_S_2_ 747.0483).

*(−)-(1S,2R,6R,8S,6’S)-Discorhabdin B dimer* (**2**): green film; [α]${}_{D}^{20}$ = −473 (*c* 0.05, MeOH); IR (film) *v*_max_ 2916, 2848, 1675, 1620, 1561, 1524, 1203, 1132 cm^−1^; ^1^H NMR (CD_3_OD, 600 MHz) and ^13^C NMR (CD_3_OD, 150 MHz, extracted from HSQC and HMBC spectra) Table 1 and Table 2; HR-ESIMS *m/z* [M]^+^ 747.0487 (calcd. for C_36_H_24_BrN_6_O_4_S_2_ 747.0483).

*(−)-(1R,2R,6R,8S,6’S)-16’,17’-Dehydrodiscorhabdin B dimer* (**3**): orange film; [α]${}_{D}^{20}$ = −239 (*c* 0.1, MeOH); IR (film) *v*_max_ 2925, 2851, 1678, 1639, 1557, 1527, 1472, 1206 cm^−1^; ^1^H NMR (CD_3_OD, 600 MHz) and ^13^C NMR (CD_3_OD, 150 MHz) Table 1 and Table 2; HR-ESIMS *m/z* [M]^+^ 745.0328 (calcd. for C_36_H_22_BrN_6_O_4_S_2_ 745.0327).

### 4.3. Anticancer and Cytotoxicity Assays

The human colon cancer cell line HCT-116 and the human keratinocyte cell line HaCaT were used for assessing anticancer activity (test concentration 100 µg/mL). Cells were supplemented at 37 °C and 5% CO_2_ in RPMI (Roswell Park memorial institute) 1640 medium (Life Technologies, Darmstadt, Germany) with 10% fetal bovine serum, 100 U/mL penicillin, and 100 mg/mL streptomycin. A stock solution of 20 mg/mL in DMSO was prepared for each test sample. After a 24 h incubation in 96 well plates, the medium in the cells was replaced by 100 µL fresh medium containing the test samples and cells were incubated for another 24 h at 37 °C. Doxorubicin was used as positive control, while 0.5% DMSO and the growth media served as negative controls. All samples were prepared in duplicates. The assay was performed according to the manufacturer’s instructions (Promega). Cells were incubated for 2 h at 37°C, and fluorescence at an excitation wavelength of 560 nm and emission at 590 nm was measured. For the determination of IC_50_ values, a dilution series of the extracts were tested following the same procedure as described before. IC_50_ values were calculated by using Excel to determine the concentration that shows a 50% inhibition of the viability.

## Figures and Tables

**Table 1:** ^1^H NMR (600 MHz) data of compounds **1**–**3** in CD_3_OD (TFA salts, *δ* in ppm).

Position	1	2	3
	*δ*_H_, Mult. (*J* in Hz)	*δ*_H_, Mult. (*J* in Hz)	*δ*_H_, Mult. (*J* in Hz)
1	4.72 d (3.1)	5.03 d (2.6)	4.53 d (3.1)
2	4.45 d (3.1)	4.43 d (2.6)	4.45 d (3.1)
4	6.17 s	6.27 s	6.04 s
7α	2.71 dd (1.3, 12.0)	2.76 dd (1.5, 11.7)	2.31 dd (1.5, 12.0)
7β	2.91 dd (3.7, 12.0)	2.95 dd (3.6, 11.7)	2.38 dd (3.7, 12.0)
8	5.62 dd (1.3, 3.7)	5.58 dd (1.5, 3.6)	5.30 dd (1.5, 3.7)
14	7.15 s	7.09 s	7.13 s
16	3.24 m	3.03 m	3.24 m
	3.10 ddd (2.6, 6.5, 16.3)		3.07 ddd (2.7, 6.8, 16.3)
17	4.13 ddd (2.6, 7.4, 13.9)	3.91 m	4.11 ddd (2.7, 7.3, 14.2)
	3.91 m		3.89 m
1’	7.89 s	7.87 (s)	7.91 s
4’	6.66 s	6.72 s	6.48 s
7’	4.79 d (7.5)	4.82 d (7.5)	4.21 d (7.5)
8’	6.57 d (7.5)	6.58 d (7.5)	6.60 d (7.5)
14’	7.25 s	7.23 s	8.15 s
16’	2.98 m	2.97 m	7.64 d (6.0)
17’	3.91 m	3.91 m	8.19 d (6.0)
TFA: Trifluoroacetic acid			

**Table 2:** ^13^C NMR (150 MHz) data of compounds **1**–**3** in CD_3_OD (TFA salts, *δ* in ppm).

Position	1	2	3
	*δ*_C_^a^	*δ*_C_^a^	*δ*_C_
1	46.0 (CH)	44.9 (CH)	45.8 (CH)
2	65.6 (CH)	68.0 (CH)	66.1 (CH)
3	182.3 (C)	183.7 (C)	182.3 (C)
4	114.4 (CH)	114.8 (CH)	114.4 (CH)
5	171.0 (C)	174.4 (C)	171.7 (C)
6	46.6 (C)	48.3 (C)	47.0 (C)
7	38.8 (CH_2_)	39.2 (CH_2_)	38.4 (CH_2_)
8	63.8 (CH)	62.8 (CH)	63.6 (CH)
10	148.6 (C)	150.4 (C)	148.4 (C)
11	167.0 (C)	167.0 (C)	/
12	125.4 (C)	125.8 (C)	125.4 (C)
14	127.5 (CH)	127.5 (CH)	127.4 (CH)
15	119.4 (C)	119.5 (C)	119.4 (C)
16	20.8 (CH_2_)	20.7 (CH_2_)	20.7 (CH_2_)
17	52.9 (CH_2_)	52.9 (CH_2_)	52.9 (CH_2_)
19	150.4 (C)	150.6 (C)	150.2 (C)
20	101.1 (C)	98.6 (C)	101.2 (C)
21	122.8 (C)	122.7 (C)	122.8 (C)
1’	150.5 (CH)	150.2 (CH)	156.0 (CH)
2’	124.3 (C)	124.4 (C)	120.0 (C)
3’	176.2 (C)	176.2 (C)	178.4 (C)
4’	122.3 (CH)	121.9 (CH)	118.7 (CH)
5’	163.2 (C)	164.7 (C)	171.4 (C)
6’	50.8 (C)	50.7 (C)	52.5 (C)
7’	115.1 (CH)	114.9 (CH)	103.9 (CH)
8’	126.9 (CH)	126.4 (CH)	128.8 (CH)
10’	146.8 (C)	147.2 (C)	142.3 (C)
11’	166.5 (C)	166.5 (C)	/
12’	125.6 (C)	125.8 (C)	120.9
14’	127.5 (CH)	127.5 (CH)	129.7 (CH)
15’	120.9 (C)	121.2 (C)	126.3 (C)
16’	19.3 (CH_2_)	19.2 (CH_2_)	115.9 (CH)
17’	46.2 (CH_2_)	46.1 (CH_2_)	142.0 (CH)
19’	159.9 (C)	160.4 (C)	148.4 (C)
20’	95.8 (C)	96.1 (C)	108.0 (C)
21’	123.2 (C)	123.2 (C)	121.0 (C)

^a^ extracted from HSQC and HMBC spectra. TFA: Trifluoroacetic acid.

**Table 3:** In vitro bioactivity and toxicity of compounds **1** and **2**.

	IC_50_ Values (μM)	
	HCT-116 Cells	HaCaT Cells
Compound **1** ^a^	0.16	0.56
Compound **2** ^a^	2.01	4.69
Negative (solvent) control ^b^	-	-
Positive control ^c^	22.1	35.1

^a^ All compounds were tested as TFA salts; ^b^ Solvent control: 0.5% DMSO; ^c^ Positive control: doxorubicin.

## Supplementary Materials

The following are available online at https://www.mdpi.com/1660-3397/18/2/107/s1, HR-ESIMS and NMR spectra of compounds **1**–**3**.
