# Supplementary material for: New Discorhabdin B Dimers with Anticancer Activity from the Antarctic Deep-Sea Sponge Latrunculia biformis
Source: Mar Drugs. 2020 Feb 11;18(2):107. doi: 10.3390/md18020107 (PMC7074271; doi:10.3390/md18020107)

SUPPLEMENTARY MATERIALS

New Discorhabdin B Dimers with Anticancer Activity from the Antarctic Deep-Sea Sponge *Latrunculia biformis*

Fengjie Li [1], Dorte Janussen [2] and Deniz Tasdemir [1,3,*]

[1] GEOMAR Centre for Marine Biotechnology (GEOMAR-Biotech), Research Unit Marine Natural Products Chemistry, GEOMAR Helmholtz Centre for Ocean Research Kiel, Am Kiel-Kanal 44, 24106, Kiel, Germany; fli@geomar.de

[2] Senckenberg Research Institute and Natural History Museum, Senckenberganlage 25, D-60325 Frankfurt, Germany; dorte.janussen@senckenberg.de

[3] Faculty of Mathematics and Natural Sciences, Kiel University, Christian-Albrechts-Platz 4, 24118 Kiel, Germany

* Correspondence: dtasdemir@geomar.de; Tel.: +49-431-600-4430; ORCID ID: 0000-0002-7841-6271

Figure **Page**

Figure S1. ^{1}H NMR spectrum of compound **1** (TFA salt, 600 MHz, CD_3OD). 2

Figure S2. HSQC spectrum of compound **1** (TFA salt, 600 MHz, CD_3OD). 2

Figure S3. HMBC spectrum of compound **1** (TFA salt, 600 MHz, CD_3OD). 3

Figure S4. COSY spectrum of compound **1** (TFA salt, 600 MHz, CD_3OD). 3

Figure S5. NOESY spectrum of compound **1** (TFA salt, 600 MHz, CD_3OD). 4

Figure S6. HR-ESIMS spectrum of compound **1**. 4

Figure S7. ^{1}H NMR spectrum of compound **2** (TFA salt, 600 MHz, CD_3OD). 5

Figure S8. HSQC spectrum of compound **2** (TFA salt, 600 MHz, CD_3OD). 5

Figure S9. HMBC spectrum of compound **2** (TFA salt, 600 MHz, CD_3OD). 6

Figure S10. COSY spectrum of compound **2** (TFA salt, 600 MHz, CD_3OD). 6

Figure S11. NOESY spectrum of compound **2** (TFA salt, 600 MHz, CD_3OD). 7

Figure S12. HR-ESIMS spectrum of compound **2**. 7

Figure S13. ^{1}H NMR spectrum of compound **3** (TFA salt, 600 MHz, CD_3OD). 8

Figure S14. ^{13}C NMR spectrum of compound **3** (TFA salt, 150MHz, CD3OD). 8

Figure S15. HSQC spectrum of compound **3** (TFA salt, 600 MHz, CD_3OD). 9

Figure S16. HMBC spectrum of compound **3** (TFA salt, 600 MHz, CD_3OD). 9

Figure S17. COSY spectrum of compound **3** (TFA salt, 600 MHz, CD_3OD). 10

Figure S18. NOESY spectrum of compound **3** (TFA salt, 600 MHz, CD_3OD). 10

Figure S19. HR-ESIMS spectrum of compound **3**. 11

Figure S1. ^{1}H NMR spectrum of compound **1** (TFA salt, 600 MHz, CD_3OD).

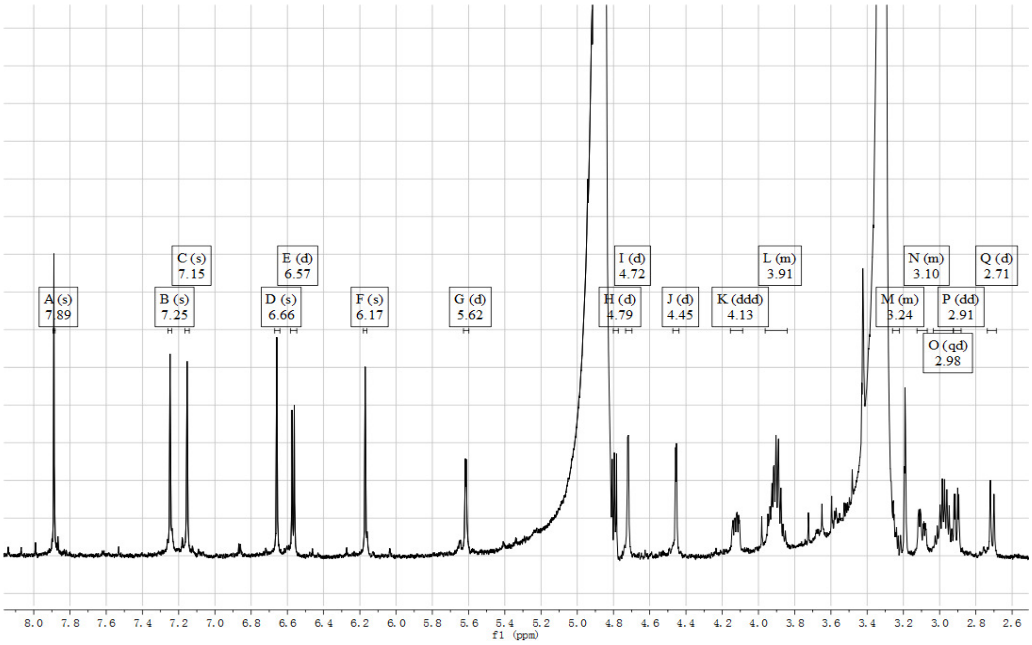

Figure S2. HSQC spectrum of compound **1** (TFA salt, 600 MHz, CD_3OD).

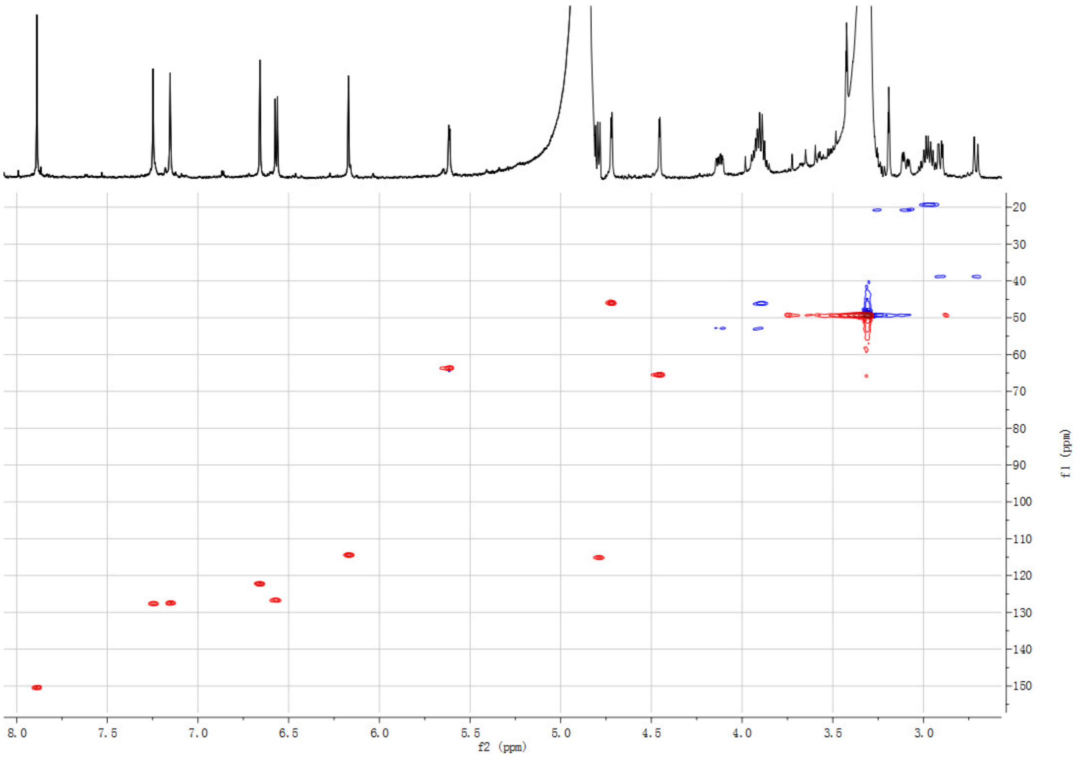

Figure S3. HMBC spectrum of compound **1** (TFA salt, 600 MHz, CD_3OD).

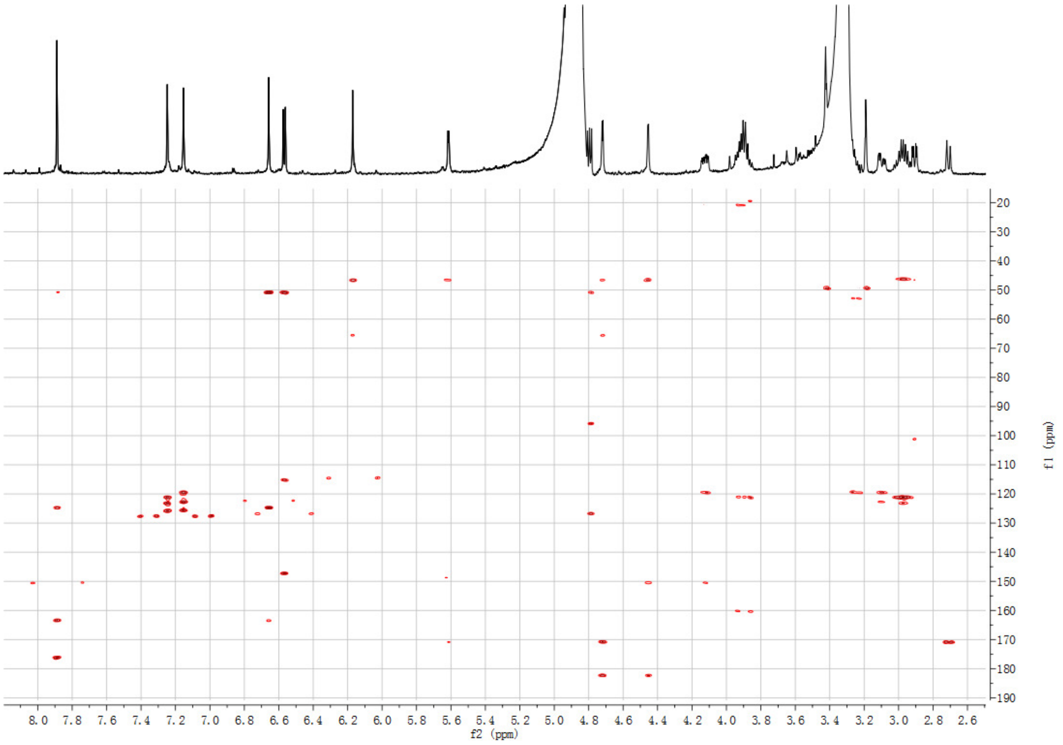

Figure S4. COSY spectrum of compound **1** (TFA salt, 600 MHz, CD_3OD).

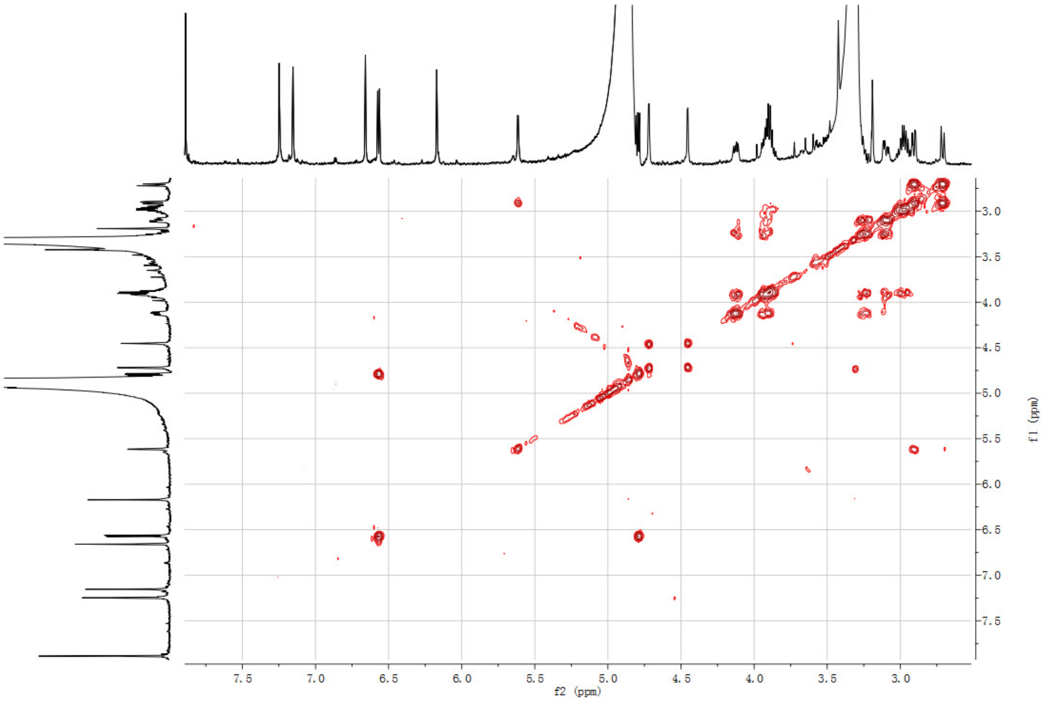

Figure S5. NOESY spectrum of compound **1** (TFA salt, 600 MHz, CD_3OD).

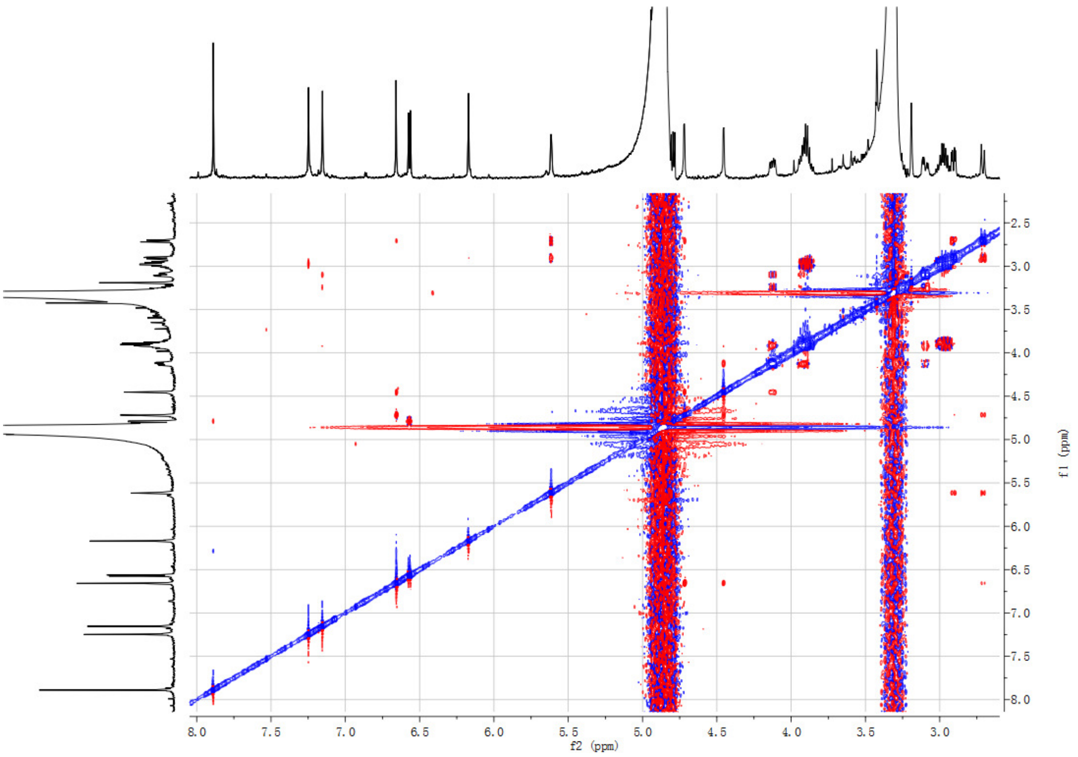

Figure S6. HR-ESIMS spectrum of compound **1**.

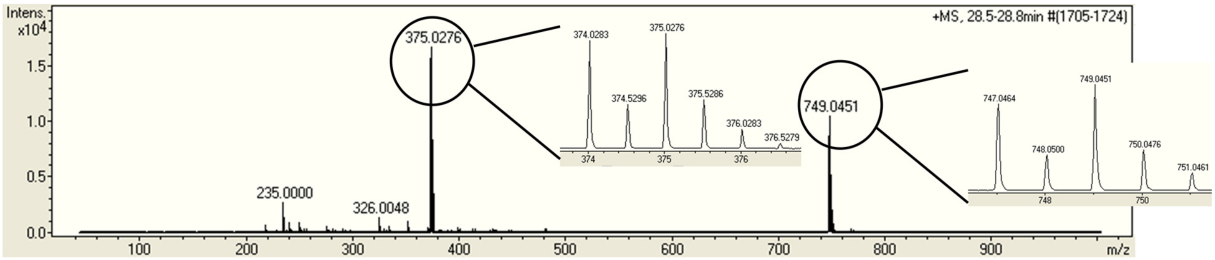

Figure S7. ^{1}H NMR spectrum of compound **2** (TFA salt, 600 MHz, CD_3OD).

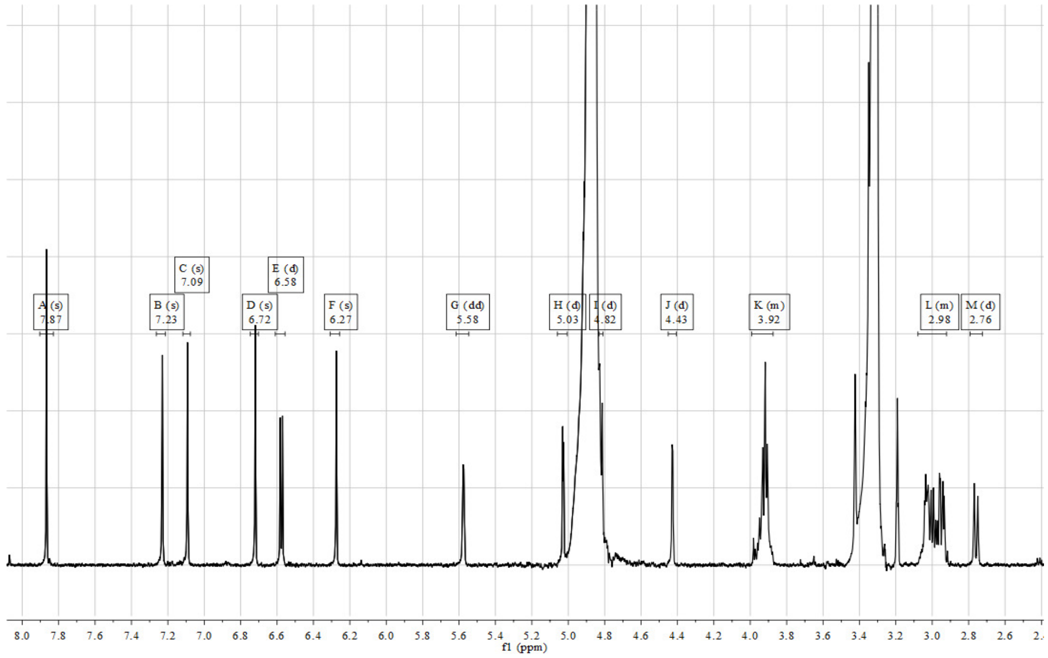

Figure S8. HSQC spectrum of compound **2** (TFA salt, 600 MHz, CD_3OD).

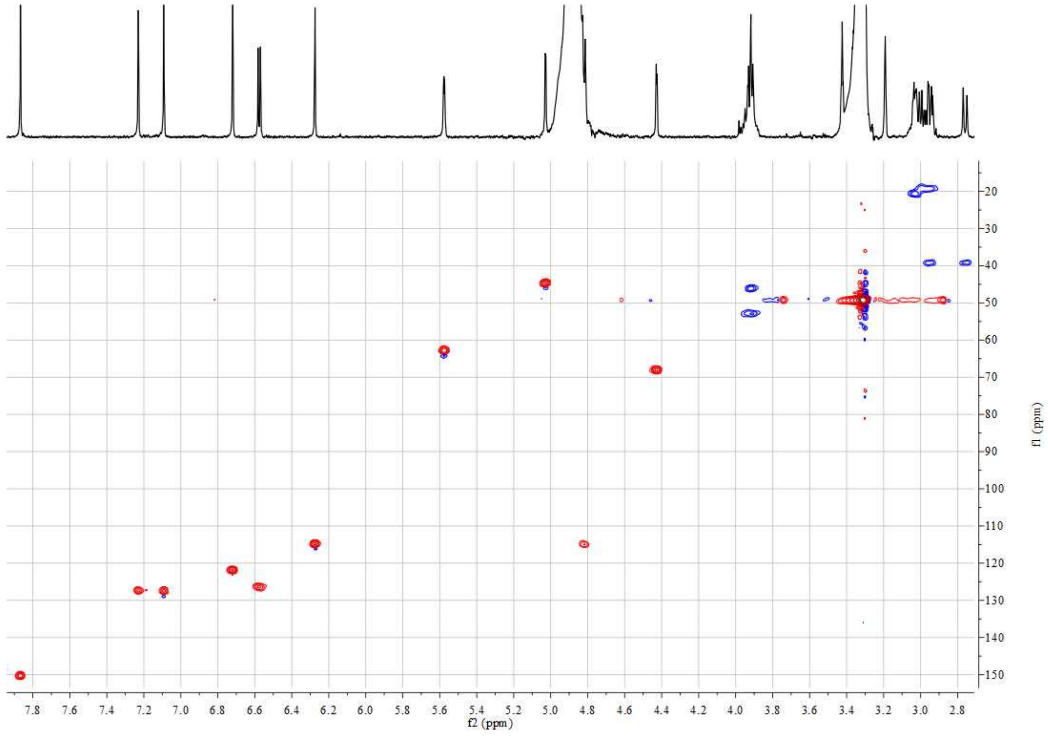

Figure S9. HMBC spectrum of compound **2** (TFA salt, 600 MHz, CD_3OD).

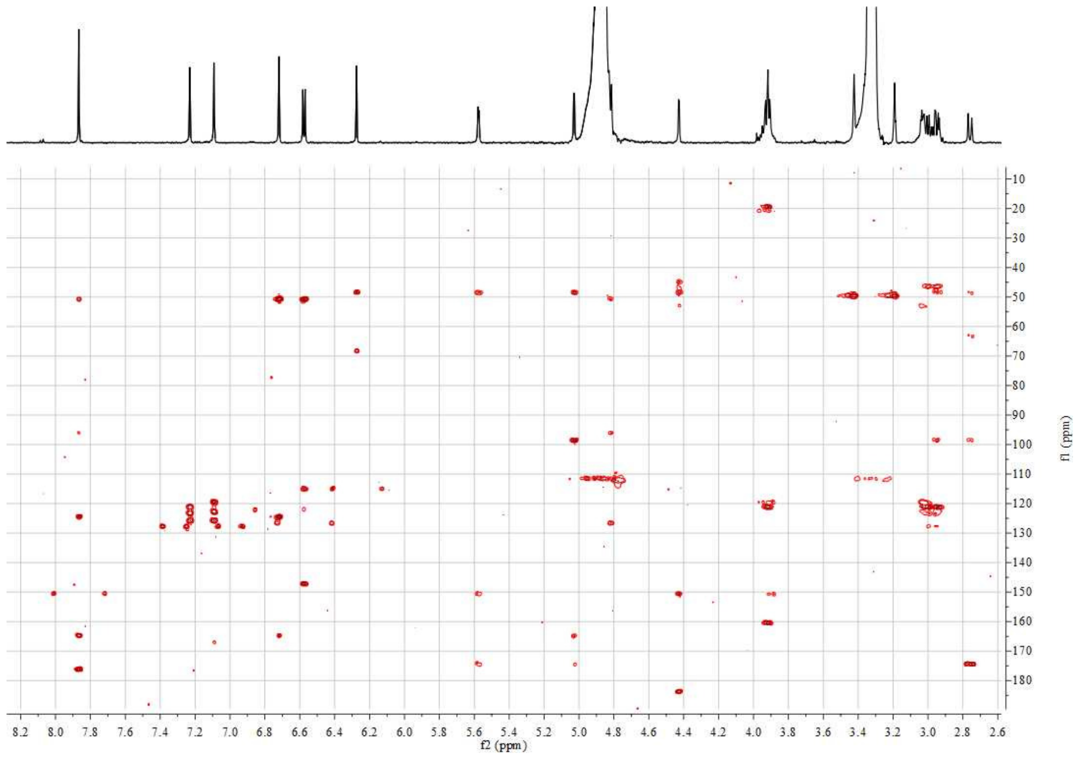

Figure S10. COSY spectrum of compound **2** (TFA salt, 600 MHz, CD_3OD).

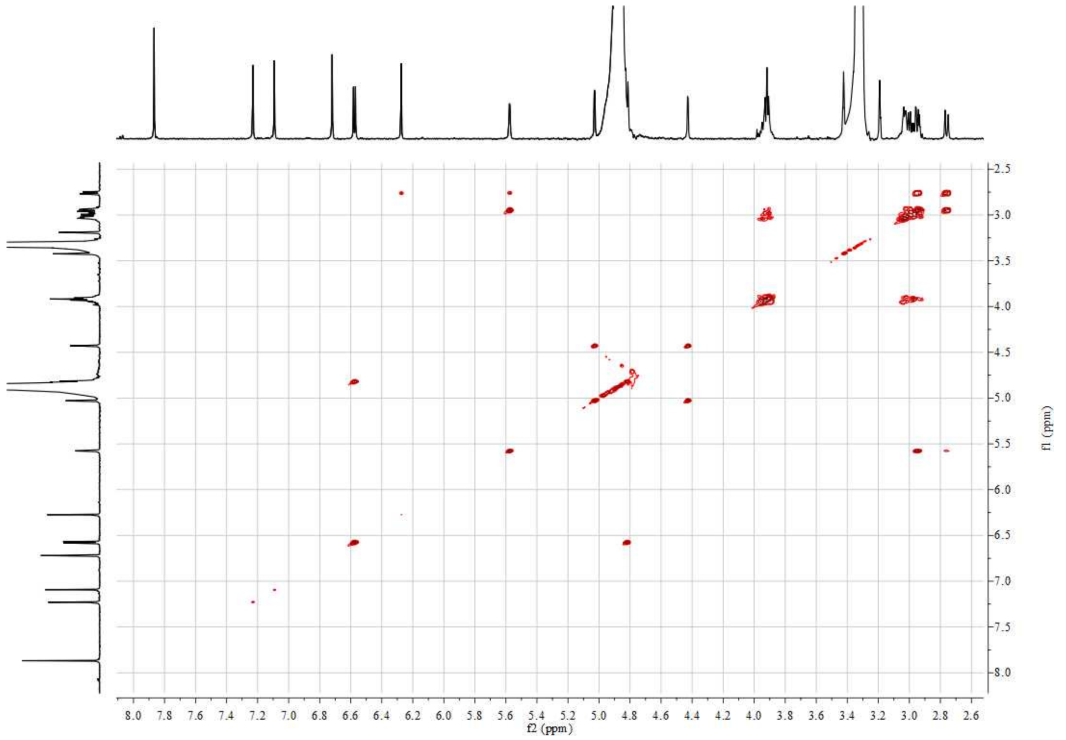

Figure S11. NOESY spectrum of compound **2** (TFA salt, 600 MHz, CD_3OD).

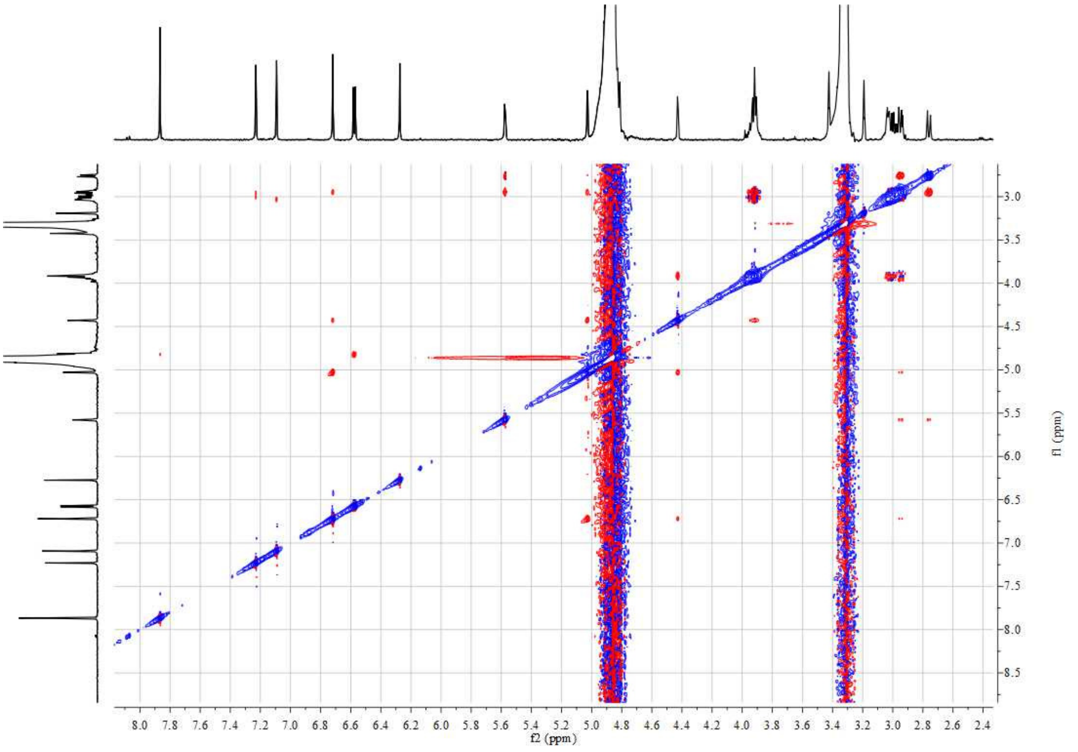

Figure S12. HR-ESIMS spectrum of compound **2**.

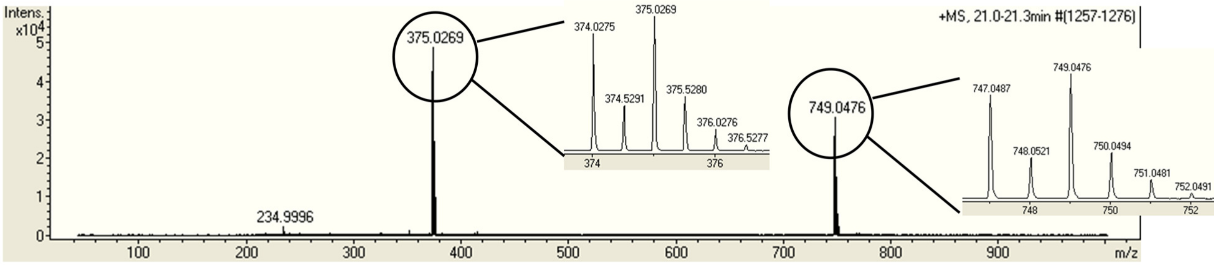

Figure S13. ^{1}H NMR spectrum of compound **3** (TFA salt, 600 MHz, CD_3OD).

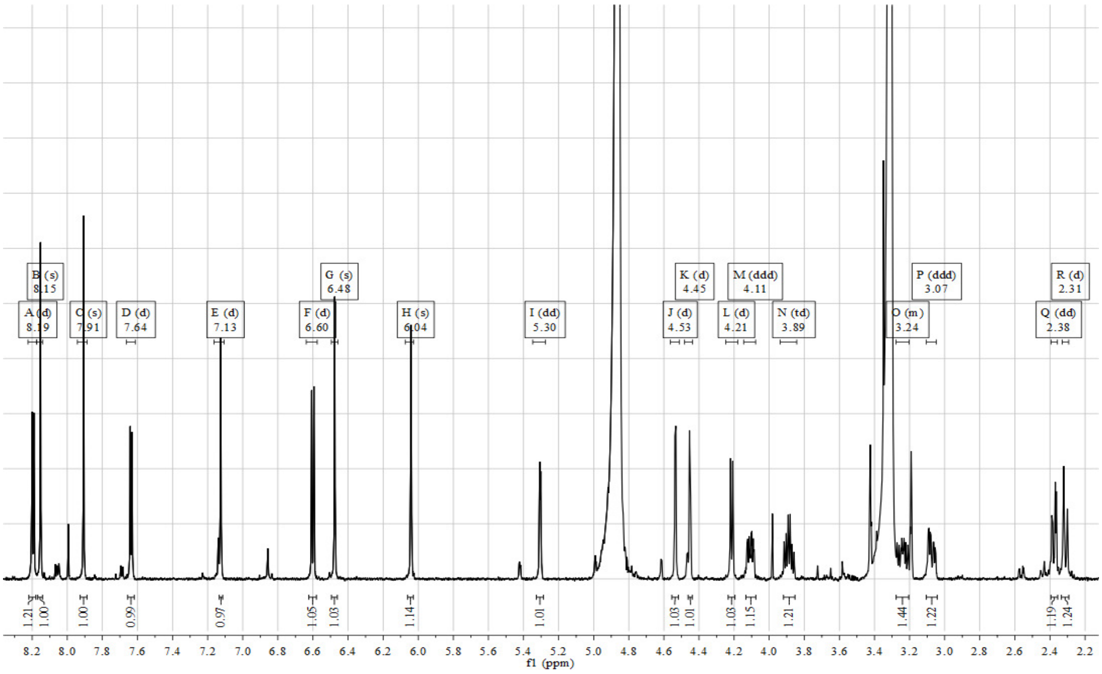

Figure S14. ^{13}C NMR spectrum of compound **3** (TFA salt, 150MHz, CD3OD).

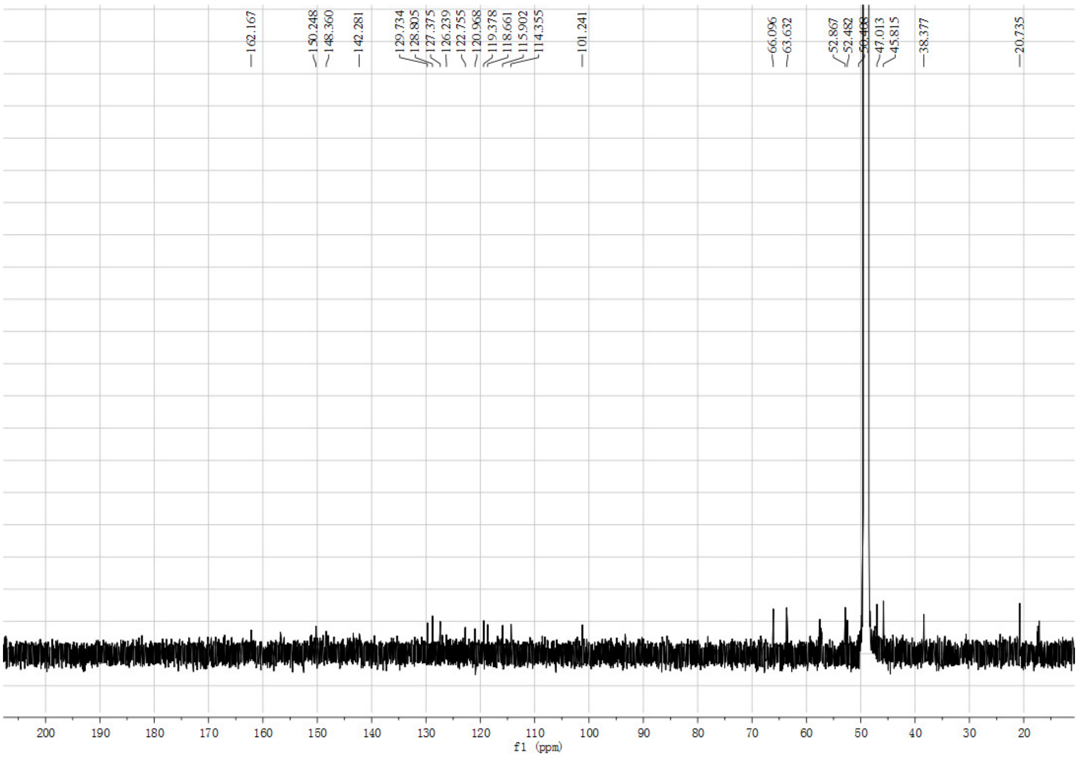

Figure S15. HSQC spectrum of compound **3** (TFA salt, 600 MHz, CD3OD).

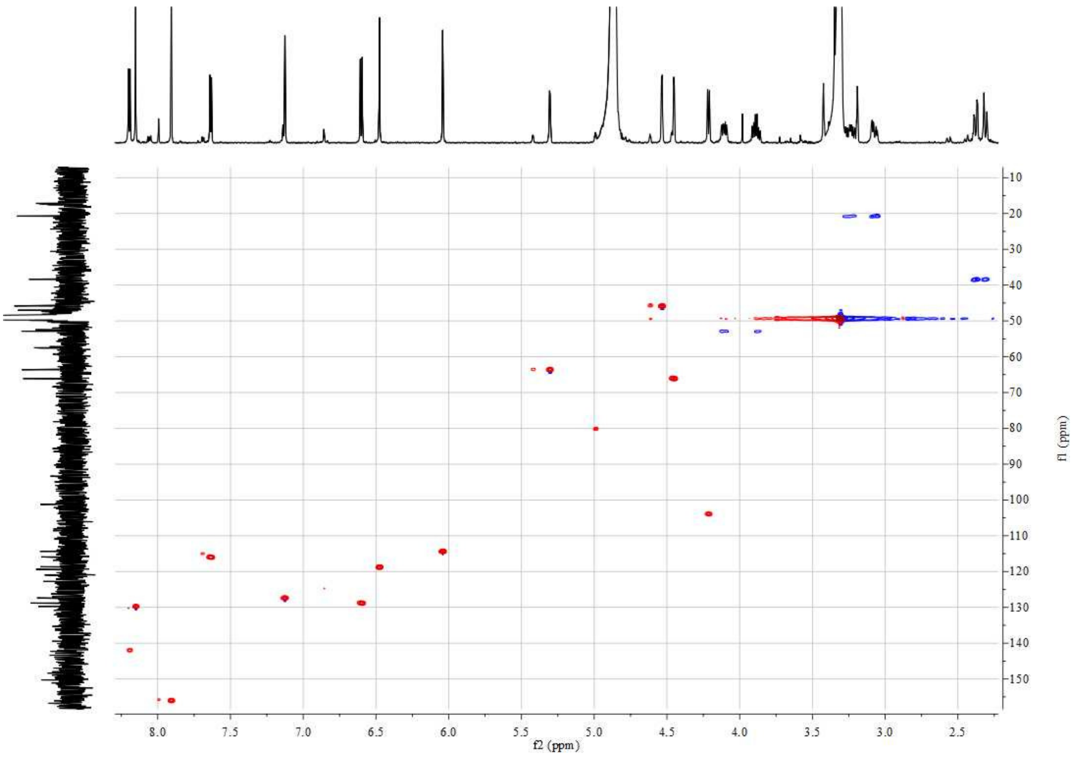

Figure S16. HMBC spectrum of compound **3** (TFA salt, 600 MHz, CD3OD).

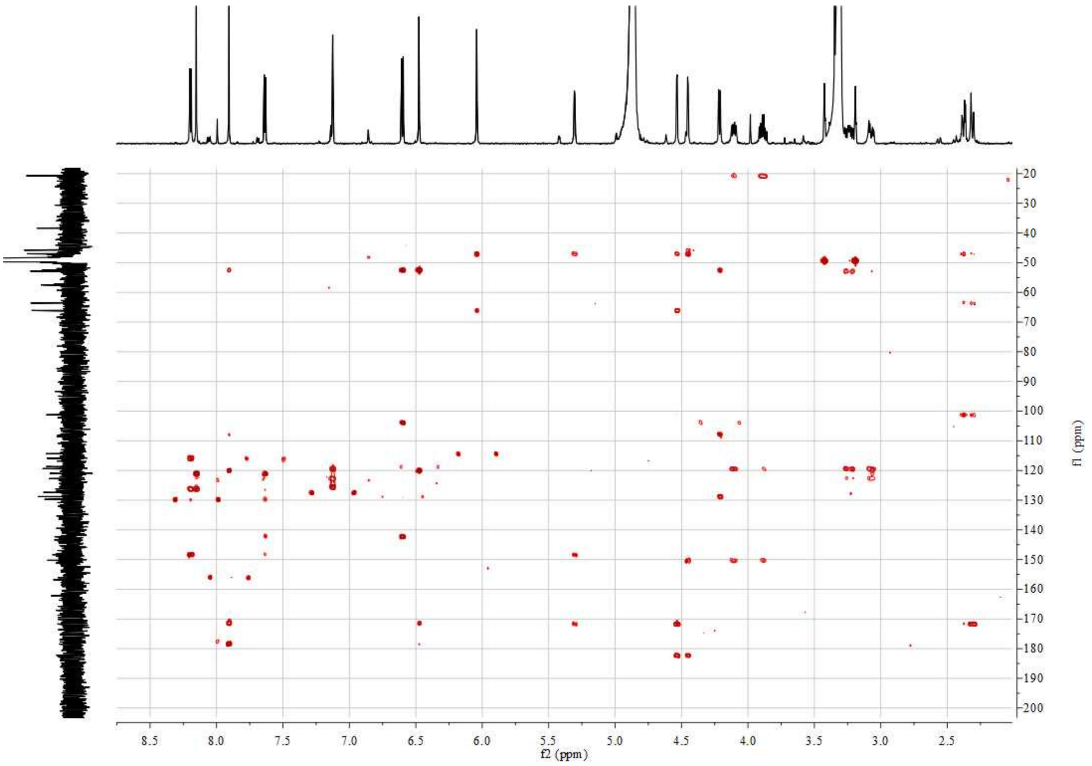

Figure S17. COSY spectrum of compound **3** (TFA salt, 600 MHz, CD_3OD).

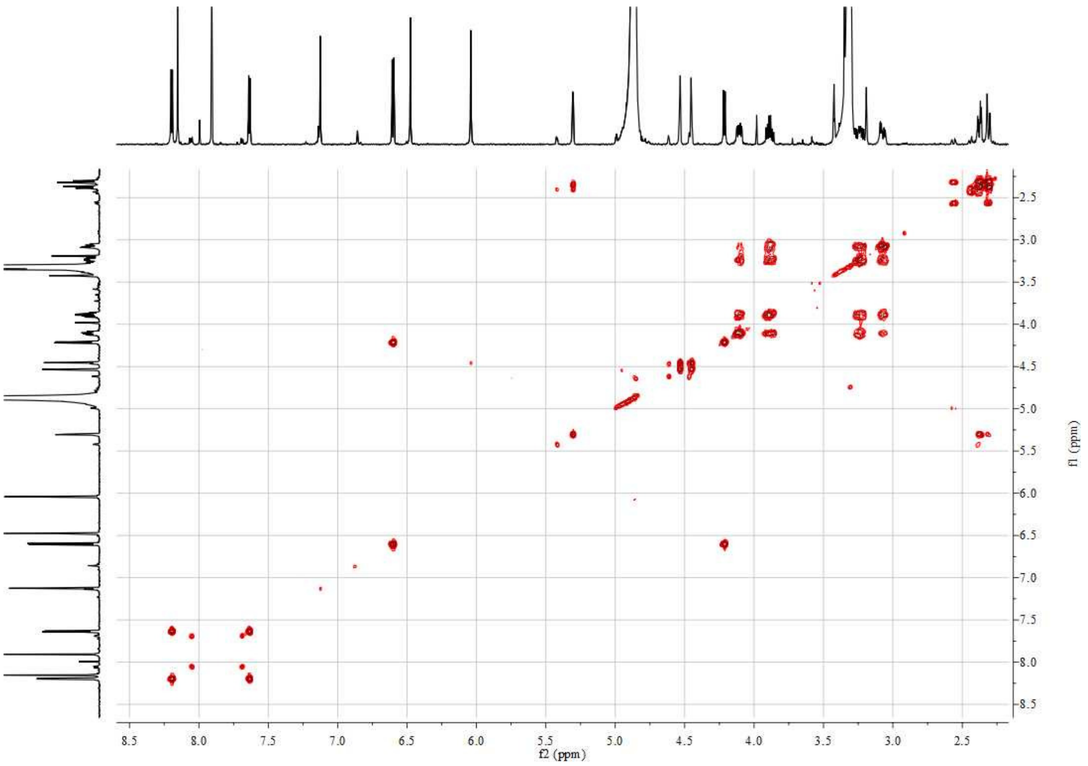

Figure S18. NOESY spectrum of compound **3** (TFA salt, 600 MHz, CD_3OD).

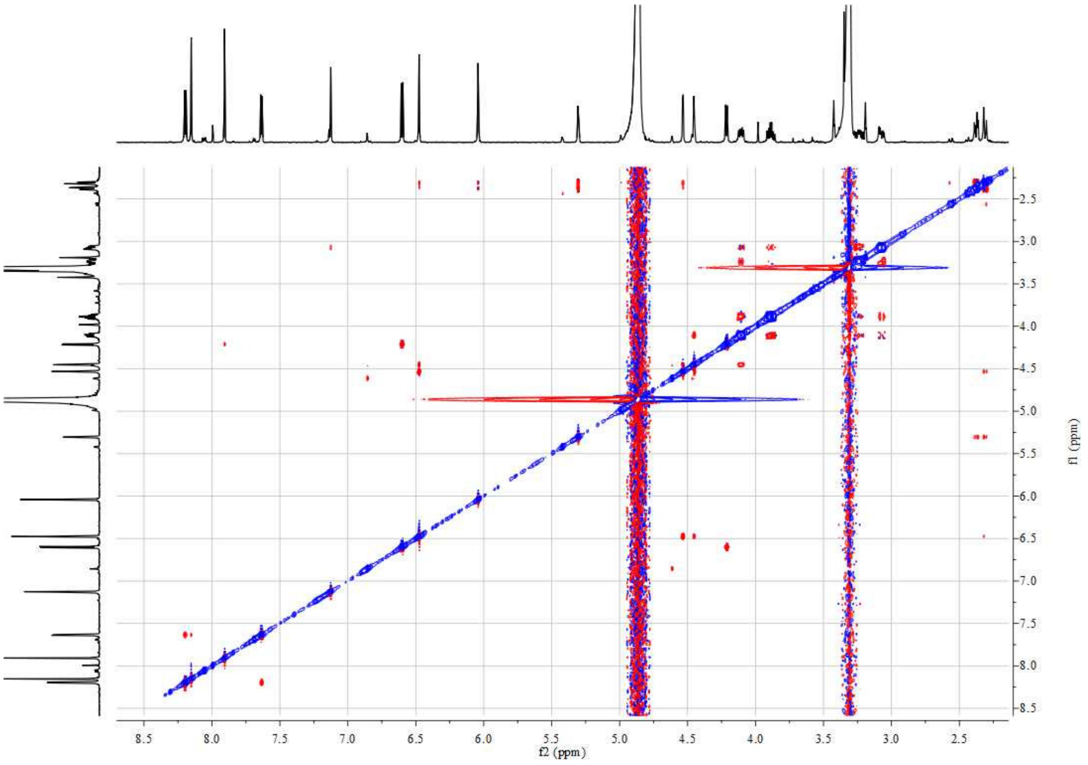

Figure S19. HR-ESIMS spectrum of compound **3**.

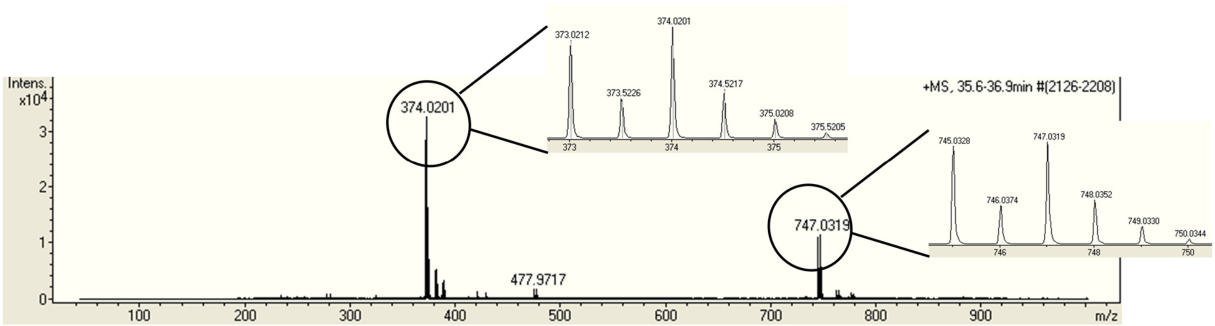

Supplement: Supplementary file 1 [file marinedrugs-18-00107-s001.pdf]
